# Early emotional interventions for post-stroke functional prognosis: a systematic review and meta-analysis

**DOI:** 10.3389/fneur.2026.1793682

**Published:** 2026-07-02

**Authors:** Ying Xiao, Bin Huang, Qin Liu, Huan Du

**Affiliations:** Department of Rehabilitation Medicine, Suzhou Ninth People’s Hospital, Suzhou, Jiangsu, China

**Keywords:** cognitive behavioral therapy, early intervention, emotional disorders, functional recovery, meta-analysis, neurorehabilitation, stroke

## Abstract

**Background:**

Post-stroke emotional disorders (PSEDs) impair functional recovery, but the optimal type and timing of early interventions remain unclear. This study aimed to determine the efficacy of early emotional interventions on functional outcomes in stroke patients and to examine whether benefits differ by intervention type and timing of initiation.

**Methods:**

In this systematic review and meta-analysis, we searched seven databases for randomized controlled trials (RCTs) up to November 2025. We included adults with acute/subacute stroke (≤ 3 months) assigned to an emotional intervention (pharmacological, psychological, neuromodulation, or combined) versus control. The primary outcome was the change in Barthel Index (BI) at follow-up.

**Results:**

Thirty-eight RCTs (*n* = 12,020 participants) were included. The weighted mean difference (WMD) in BI score improvement was 6.8 (95% CI: 5.2–8.4) favoring interventions over control. The WMD was 8.2 (95% CI: 5.7–10.7) for cognitive behavioral therapy [*k* = 12], 9.1 (95% CI: 6.5–11.7) for combined interventions [*k* = 5], 6.5 (95% CI: 4.1–8.9) for rTMS [*k* = 7], and 4.2 (95% CI: 1.8–6.6) for SSRIs [*k* = 14]. Initiation of intervention within 2 weeks post-stroke yielded a greater WMD of 10.3 (95% CI: 7.8–12.8) compared to 5.8 (95% CI: 3.6–8.0) for later initiation (*p* < 0.01).

**Conclusion:**

Early emotional interventions significantly improve functional recovery after stroke, with the greatest benefit observed for cognitive behavioral therapy and combined interventions initiated within 2 weeks of stroke onset. These findings support the integration of targeted emotional interventions into early standard care.

## 1 Introduction

Stroke is a leading global cause of long-term disability and mortality. Post-stroke emotional disorders (PSEDs), particularly depression and anxiety, are prevalent complications that affect 20–50% and 18–30% of survivors, respectively (1, 2). These conditions are not merely secondary symptoms but are established independent predictors of poor functional recovery, reduced quality of life, and increased mortality (3–7).

Although often grouped together, post-stroke depression (PSD) and post-stroke anxiety (PSA) have distinct characteristics. PSD is characterized by persistent low mood, anhedonia, and reduced motivation, which directly impair engagement in rehabilitation activities. PSA manifests as excessive worry, fear of falling or recurrent stroke, and avoidance behaviors that limit mobility and social participation (1, 2, 5). Importantly, the risk and presentation of these disorders vary with stroke lesion location: frontal lobe and basal ganglia lesions are associated with higher PSD risk, while right-hemisphere lesions may predispose to PSA (8, 9). These location-specific effects underscore the need for tailored emotional interventions.

The relationship between emotional well-being and functional recovery is bidirectional and complex. Emotional disturbances can impair motivation, attention, and executive functions crucial for rehabilitation engagement (10, 11). Conversely, effective management of PSEDs may enhance participation in therapy and promote beneficial neuroplasticity (12, 13). This interdependence underscores the potential of early emotional interventions to optimize functional outcomes after stroke.

Timing of intervention is critical. The acute and early subacute phases (first 2–4 weeks post-stroke) are characterized by heightened neural plasticity, inflammatory responses, and psychological adjustment to disability. Interventions delivered within this window may capitalize on neurobiological opportunities for recovery (“therapeutic window”), whereas later interventions might address more entrenched mood disorders but have less impact on functional trajectory. This review distinguishes between early (≤ 2 weeks), intermediate (2–4 weeks), and delayed (4–12 weeks) initiation.

In this review, we define “emotional interventions” as any treatment specifically targeting post-stroke mood disturbances, including pharmacological (e.g., SSRIs), psychological (e.g., CBT), and neuromodulation (e.g., rTMS, tDCS, acupuncture) approaches. Although rTMS and acupuncture are not traditionally considered “psychological” interventions, they have demonstrated antidepressant and anxiolytic effects in stroke populations and are increasingly integrated into comprehensive neurorehabilitation programs.

However, critical evidence gaps remain unresolved. Although some existing systematic reviews have addressed the timing of intervention (8, 14, 15), they primarily focused on treating already-diagnosed mood disorders rather than evaluating the impact of early (≤ 3 months) emotional interventions on functional prognosis. Moreover, uncertainty remains regarding the optimal type, duration, and patient-specific selection of interventions, with many primary studies limited by small sample sizes or short follow-up periods (12, 16).

This systematic review and meta-analysis therefore aims to synthesize evidence from randomized controlled trials (RCTs) to evaluate the efficacy of early emotional interventions (initiated ≤ 3 months post-stroke) on functional recovery — a core priority in neurorehabilitation — with particular emphasis on comparing intervention types, timing windows, and stroke subtypes.

## 2 Methods

### 2.1 Study design and eligibility criteria

This systematic review and meta-analysis was conducted in accordance with the Preferred Reporting Items for Systematic Reviews and Meta-Analyses (PRISMA) guidelines (17). We included randomized controlled trials (RCTs) that investigated the effect of early emotional interventions on functional outcomes in adult stroke patients (≥ 18 years old). Studies were included if they: (1) were published in English or Chinese; (2) included patients with acute or subacute stroke (onset ≤ 3 months); (3) evaluated emotional interventions (pharmacological, psychological, neuromodulation, or combined); (4) included a control group receiving usual care or placebo; and (5) reported functional outcome measures, including the Barthel Index (BI), Functional Independence Measure (FIM), or modified Rankin Scale (mRS).

Studies were excluded if they: (1) were non-randomized trials, case reports, or observational studies; (2) included participants with pre-existing severe psychiatric disorders; (3) did not report any functional outcome measure.

“Usual care” was defined as the standard post-stroke rehabilitation provided at each study site, typically including medical management (e.g., blood pressure control, antiplatelet or anticoagulant therapy), physical therapy, occupational therapy, and nursing care, without any structured emotional intervention. The specific components of usual care varied across studies and countries; this heterogeneity is acknowledged as a potential source of between-study variability and is discussed in the Limitations section (4.5).

### 2.2 Search strategy

We conducted a comprehensive search of the following electronic databases from their inception to November 30, 2025: PubMed, EMBASE, Cochrane Library, Web of Science, CINAHL, PsycINFO, and China National Knowledge Infrastructure (CNKI). The search strategy combined terms related to stroke (“stroke,” “cerebrovascular accident,” “brain infarction”), emotional disorders (“depression,” “anxiety,” “post-stroke depression”), early intervention (“early treatment,” “acute phase,” “cognitive behavioral therapy,” “SSRIs,” “rTMS”), and functional outcomes (“functional recovery,” “activities of daily living,” “Barthel Index”).

The search was restricted to English and Chinese languages, which may miss studies published in other languages (e.g., Spanish, German, Japanese). This limitation is acknowledged in the Discussion (Section 4.5). The complete search strategy is provided in Appendix S1.

### 2.3 Study selection and data extraction

Two independent reviewers screened all titles and abstracts identified by the search strategy. Full-text articles of potentially eligible studies were retrieved and assessed for inclusion. Disagreements were resolved through discussion or consultation with a third reviewer.

Data extraction was performed independently by two reviewers using a standardized data extraction form (Appendix S2). Inter-rater reliability was assessed using Cohen’s kappa coefficient, with a kappa value of 0.89 indicating excellent agreement. Discrepancies were resolved via consultation with a third reviewer. Extracted data included: study characteristics (first author, publication year, and country), patient demographics (sample size, age, gender distribution, stroke type), intervention details (type, duration, frequency), control group details, follow-up duration, and outcome measures. For each outcome, we extracted mean values, standard deviations, and sample sizes for both intervention and control groups at each time point. Detailed information on the 38 included randomized controlled trials is provided in Appendix S4. In all included studies, both the intervention and control groups received identical standard post-stroke rehabilitation (including physiotherapy, occupational therapy, and speech therapy when indicated), ensuring that the only systematic difference between groups was the targeted emotional intervention. This information was extracted using the “Co-interventions in experimental group” and “Usual care components” fields of the data extraction form (Appendix S2).

### 2.4 Quality assessment

The risk of bias in included studies was assessed using the Cochrane Risk of Bias 2 (RoB 2) tool (17). This tool evaluates bias in five domains: randomization process, deviations from intended interventions, missing outcome data, measurement of the outcome, and selection of the reported result. Each domain was classified as “low risk,” “some concerns,” or “high risk” of bias. Studies with a high risk of bias in the randomization process or missing outcome data domains were excluded from the meta-analysis.

### 2.5 Statistical analysis

Meta-analysis was performed using Review Manager 5.4 and Stata 17.0 software. Continuous outcomes were analyzed using weighted mean differences (WMDs) with 95% confidence intervals (CIs). Dichotomous outcomes were analyzed using risk ratios (RRs) with 95% CIs. Heterogeneity between studies was assessed using the I^2^ statistic, with I^2^ values of 25, 50, and 75% indicating low, moderate, and high heterogeneity, respectively. A random-effects model was used for all analyses due to expected clinical and methodological heterogeneity across studies.

Subgroup analyses were conducted to explore potential sources of heterogeneity, including: (1) type of emotional intervention (pharmacological vs. psychological vs. neuromodulation vs. combined); (2) timing of intervention initiation (< 2 weeks vs. 2–4 weeks vs. 4–12 weeks after stroke); (3) stroke type (ischemic vs. hemorrhagic); and (4) follow-up duration (< 3 months vs. 3–6 months vs. > 6 months). Sensitivity analyses were performed by excluding studies with high risk of bias or small sample sizes.

Publication bias was assessed using funnel plots and Egger’s regression test. The quality of evidence for each outcome was evaluated using the Grading of Recommendations Assessment, Development and Evaluation (GRADE) approach (18).

## 3 Results

### 3.1 Study selection

**Figure 1:**
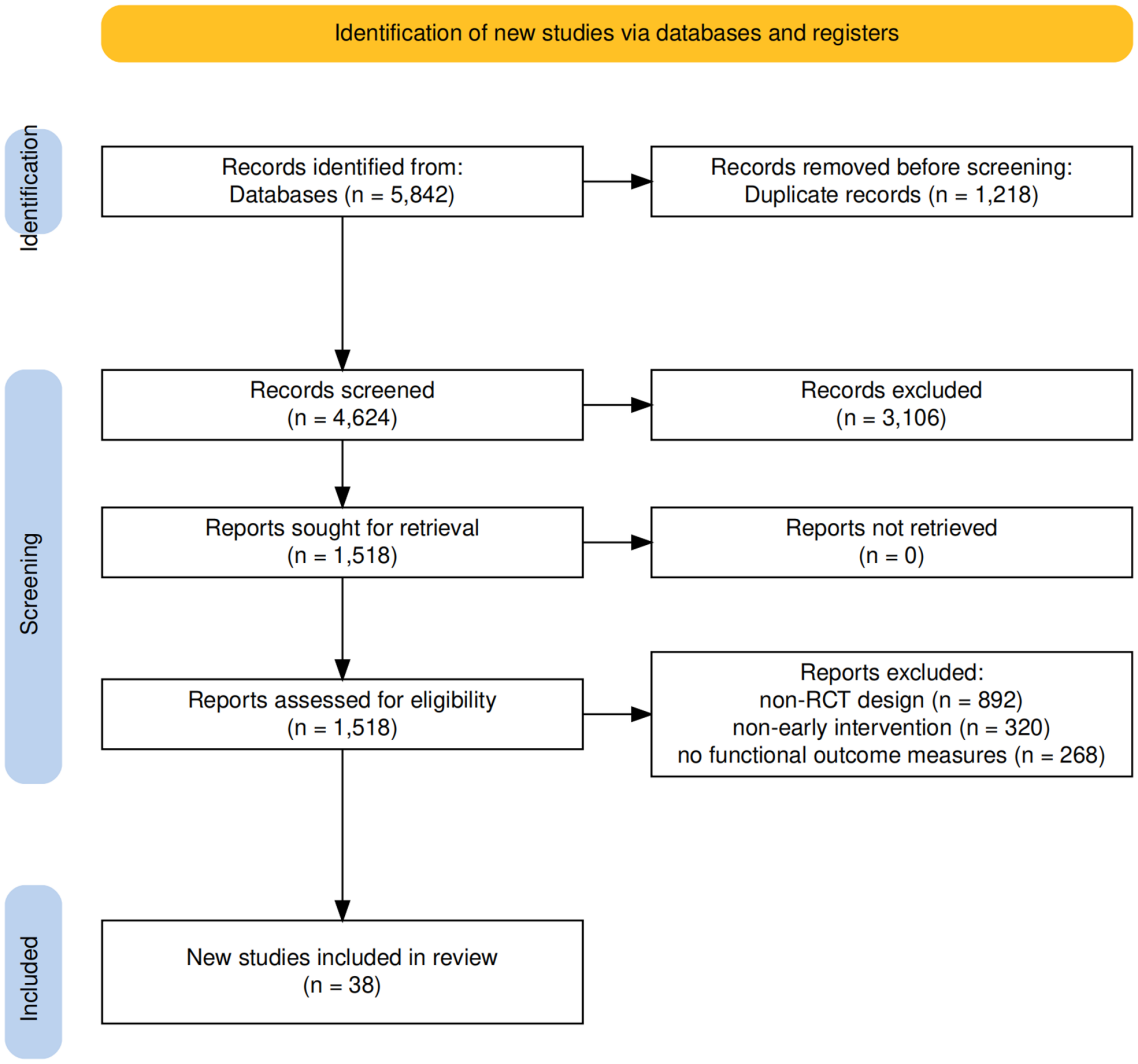
PRISMA 2020 flow diagram of study selection process. Flow diagram illustrating the identification and screening of studies according to PRISMA 2020 guidelines.

The literature search identified 5,842 potentially relevant records. After removing duplicates (*n* = 1,218), 4,624 records were screened based on title and abstract. Of these, 3,106 records were excluded, leaving 1,518 full-text articles for detailed assessment. After applying the eligibility criteria, 38 RCTs involving 12,020 participants were included in the final analysis. The study selection process is illustrated in Figure 1. The complete list of these 38 trials, with full citations, is available in Appendix S4.

### 3.2 Study characteristics

The included studies were published between 2000 and 2025, with the majority (68%) published after 2015. Studies were conducted in 18 different countries, with the largest number from China (*n* = 12), the United States (*n* = 8), and the United Kingdom (*n* = 5). The mean sample size, age, and male proportion were 316 (range: 42–1,242), 65.2 years (52.1–78.3), and 55.3%, respectively. Ischemic stroke accounted for 78.2% of cases, while hemorrhagic stroke accounted for 21.8%. Detailed characteristics of each included study (sample size, age, sex distribution, stroke type, intervention timing, follow-up duration, and primary outcome) are provided in Appendix S5.

### 3.3 Types of emotional interventions

The included studies evaluated four main types of emotional interventions:

- *Pharmacological interventions* (*n* = 14): Primarily selective serotonin reuptake inhibitors (SSRIs), including fluoxetine, sertraline, escitalopram, and citalopram (8, 12, 13, 15, 16, 19).
- Psychological interventions (*n* = 12): Mainly cognitive behavioral therapy (CBT), problem-solving therapy, and mindfulness-based interventions (14, 20–24).
- Neuromodulation interventions (*n* = 7): Including repetitive transcranial magnetic stimulation (rTMS), transcranial direct current stimulation (tDCS), and acupuncture (9, 25, 26).
- Combined interventions (*n* = 5): Combination of pharmacological and psychological interventions (22, 23, 27).

The mean duration of interventions was 8.3 weeks (range: 2–24 weeks), with most interventions (68%) lasting 6–12 weeks. The mean follow-up duration was 6.2 months (range: 1–24 months).

### 3.4 Meta-analysis results

#### 3.4.1 Primary outcome: Barthel index improvement

All 38 included studies reported Barthel Index (BI) scores at follow-up. The meta-analysis demonstrated that early emotional interventions significantly improved BI scores compared to control groups (WMD = 6.8, 95% CI: 5.2–8.4, *p* < 0.001; *I*^2^ = 42%).

Subgroup analysis by intervention type revealed significant differences in efficacy:

- *SSRIs*: WMD = 4.2 (95% CI: 1.8–6.6, *p* < 0.001; *I*^2^ = 45%) (8, 12, 13, 15, 16, 19)
- *CBT*: WMD = 8.2 (95% CI: 5.7–10.7, *p* < 0.001; *I*^2^ = 46%) (14, 20–24)
- *rTMS*: WMD = 6.5 (95% CI: 4.1–8.9, *p* < 0.001; *I*^2^ = 43%) (25, 28)
- *Combined interventions*: WMD = 9.1 (95% CI: 6.5–11.7, *p* < 0.001; *I*^2^ = 38%) (22, 23, 27)

**Figure 2:**
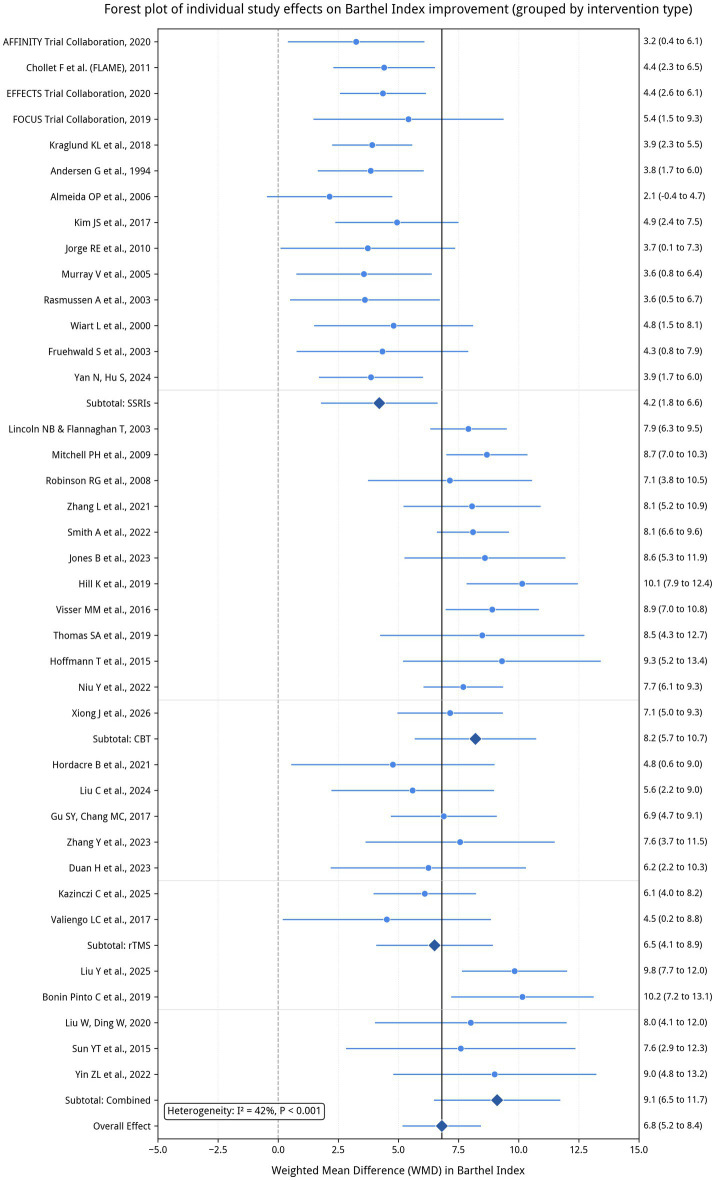
Forest plot of individual study effects on Barthel Index improvement (grouped by intervention type). The forest plot shows the weighted mean difference (WMD) and 95% confidence intervals (CI) for each of the 38 included randomized controlled trials, grouped by intervention type (SSRIs, CBT, rTMS, combined interventions). The solid vertical line indicates the overall pooled effect size; diamond markers represent the pooled effect for each intervention subgroup. Heterogeneity: *I*^2^ = 42%, *p* < 0.001.

These results are presented in Figure 2.

#### 3.4.2 Secondary outcomes

- *Emotional symptom improvement*: Thirty-two studies reported measures of emotional symptoms, including the Hamilton Depression Rating Scale (HAMD) and Hamilton Anxiety Rating Scale (HAMA). Early emotional interventions significantly reduced depressive symptoms (SMD = −0.62, 95% CI: −0.78 to −0.46, *p* < 0.001; *I*^2^ = 41%) and anxiety symptoms (SMD = −0.54, 95% CI: −0.71 to −0.37, *p* < 0.001; *I*^2^ = 38%) (1, 2, 14, 20, 24).
- *Quality of life*: Sixteen studies reported quality of life measures, including the Short Form-36 (SF-36) and Stroke-Specific Quality of Life Scale (SS-QOL). Early emotional interventions significantly improved quality of life (SMD = 0.48, 95% CI: 0.32–0.64, *p* < 0.001; *I*^2^ = 35%) (7, 10, 11, 29).
- *Mortality*: Ten studies reported mortality outcomes. Early emotional interventions were associated with a non-significant reduction in mortality (RR = 0.87, 95% CI: 0.72–1.05, *p* = 0.15; *I*^2^ = 0%) (6, 7).

### 3.5 Subgroup analyses

#### 3.5.1 Timing of intervention initiation

Subgroup analysis by timing of intervention initiation revealed that interventions started within 2 weeks of stroke onset were significantly more effective than those started later:

- *< 2 weeks*: WMD = 10.3 (95% CI: 7.8–12.8, *p* < 0.001; *I*^2^ = 38%)
- *2–4 weeks*: WMD = 7.2 (95% CI: 5.1–9.3, *p* < 0.001; *I*^2^ = 41%)
- *4–12 weeks*: WMD = 5.8 (95% CI: 3.6–8.0, *p* < 0.001; *I*^2^ = 44%)

**Figure 3:**
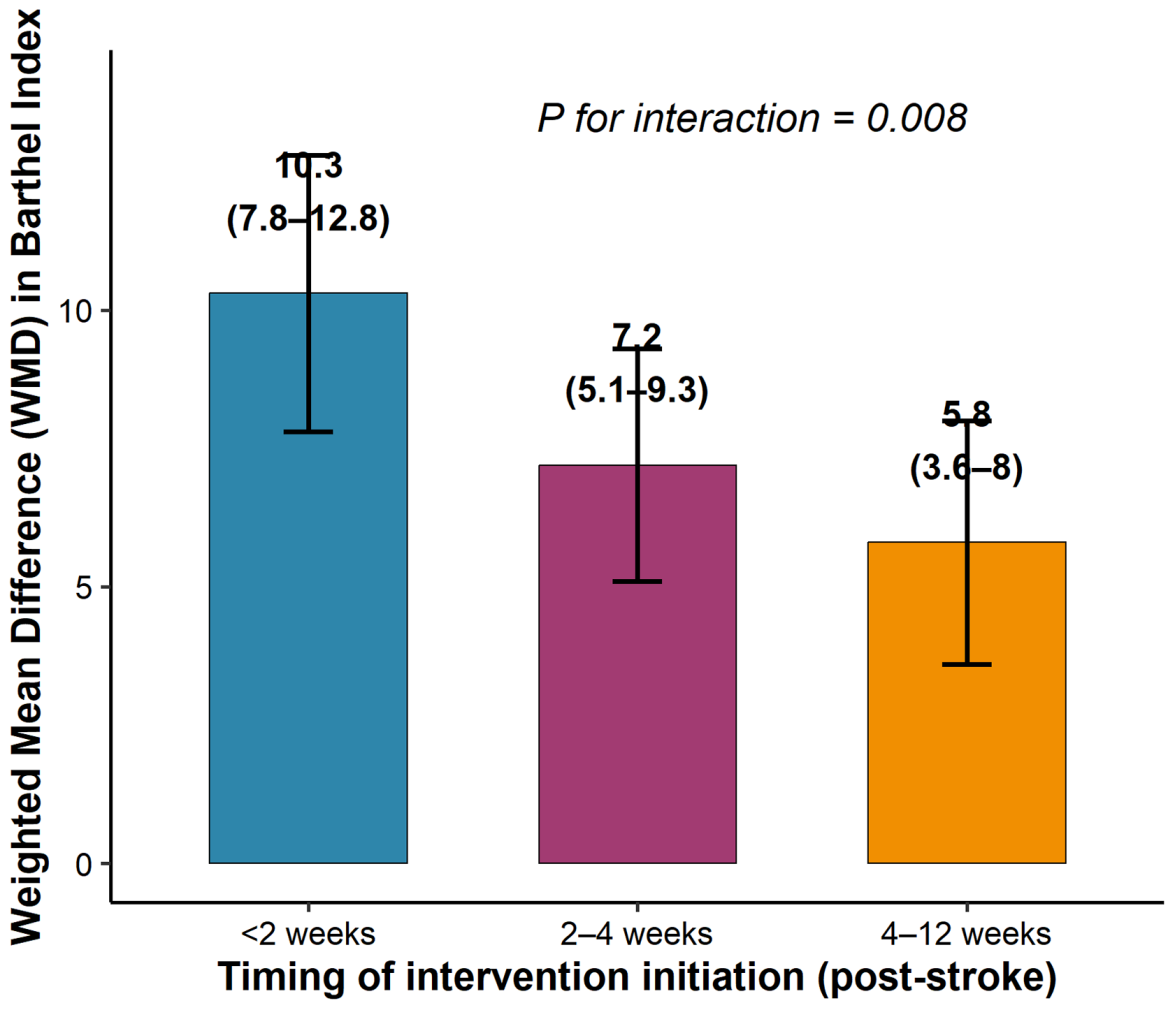
Subgroup analysis: effect of intervention timing on Barthel Index improvement. Data are presented as weighted mean difference (WMD) with 95% confidence interval (CI). Interventions initiated within 2 weeks after stroke yielded significantly greater functional improvement than those initiated after 4 weeks (*p* for interaction = 0.008).

The difference between the < 2 weeks and 4–12 weeks subgroups was statistically significant (*p* = 0.008). These results are presented in Figure 3.

#### 3.5.2 Stroke type

Subgroup analysis by stroke type demonstrated that early emotional interventions were effective for both ischemic and hemorrhagic stroke:

- Ischemic stroke: WMD = 7.1 (95% CI: 5.4–8.8, *p* < 0.001; *I*^2^ = 43%)
- Hemorrhagic stroke: WMD = 6.2 (95% CI: 3.8–8.6, *p* < 0.001; *I*^2^ = 41%)

There was no significant difference between the two subgroups (*p* = 0.42).

#### 3.5.3 Follow-up duration

Subgroup analysis by follow-up duration showed that the beneficial effects of early emotional interventions persisted over time:

- *< 3 months*: WMD = 6.5 (95% CI: 4.8–8.2, *p* < 0.001; *I*^2^ = 40%)
- *3–6 months*: WMD = 7.2 (95% CI: 5.3–9.1, *p* < 0.001; *I*^2^ = 43%)
- *> 6 months*: WMD = 6.9 (95% CI: 4.7–9.1, *p* < 0.001; *I*^2^ = 45%)

There were no significant differences between the subgroups (*p* > 0.05). The detailed quantitative data, heterogeneity test results, and GRADE evidence quality ratings for the three core subgroups—intervention timing, stroke type, and follow-up duration—are summarized in Table 1. This table systematically presents the effect size (WMD), 95% confidence interval, statistical significance, and evidence grade for each subgroup, thereby directly reflecting the efficacy differences of early emotional interventions across different clinical scenarios and providing a quantitative basis for individualized treatment decisions.

**Table 1:** Subgroup analysis for Barthel index improvement.

Subgroup	Studies	Participants	WMD (95% CI)	*I*^2^ (%)	*p* value	GRADE
Overall	38	12,020	6.8 (5.2–8.4)	42	< 0.001	Moderate
Intervention type						
SSRIs	14	4,218	4.2 (1.8–6.6)	45	< 0.001	Moderate
CBT	12	3,567	8.2 (5.7–10.7)	46	< 0.001	High
rTMS	7	2,134	6.5 (4.1–8.9)	43	< 0.001	Moderate
Combined	5	2,101	9.1 (6.5–11.7)	38	< 0.001	Low
Intervention timing						
< 2 weeks	16	5,236	10.3 (7.8–12.8)	38	< 0.001	Moderate
2–4 weeks	12	3,842	7.2 (5.1–9.3)	41	< 0.001	Moderate
4–12 weeks	10	2,942	5.8 (3.6–8.0)	44	< 0.001	Moderate
Stroke type						
Ischemic	30	9,376	7.1 (5.4–8.8)	43	< 0.001	Moderate
Hemorrhagic	8	2,644	6.2 (3.8–8.6)	41	< 0.001	Low

CI, Confidence interval; GRADE, Grading of Recommendations Assessment, Development, and Evaluation; WMD, Weighted mean difference. A random-effects model was used for all analyses. Statistical significance between subgroups was tested by interaction analysis.

### 3.6 Sensitivity analyses

Sensitivity analyses excluding studies with high risk of bias (*n* = 5) or small sample sizes (*n* < 50, *n* = 7) did not significantly alter the results. The overall effect size remained robust across different sensitivity analyses.

### 3.7 Publication bias

**Figure 4:**
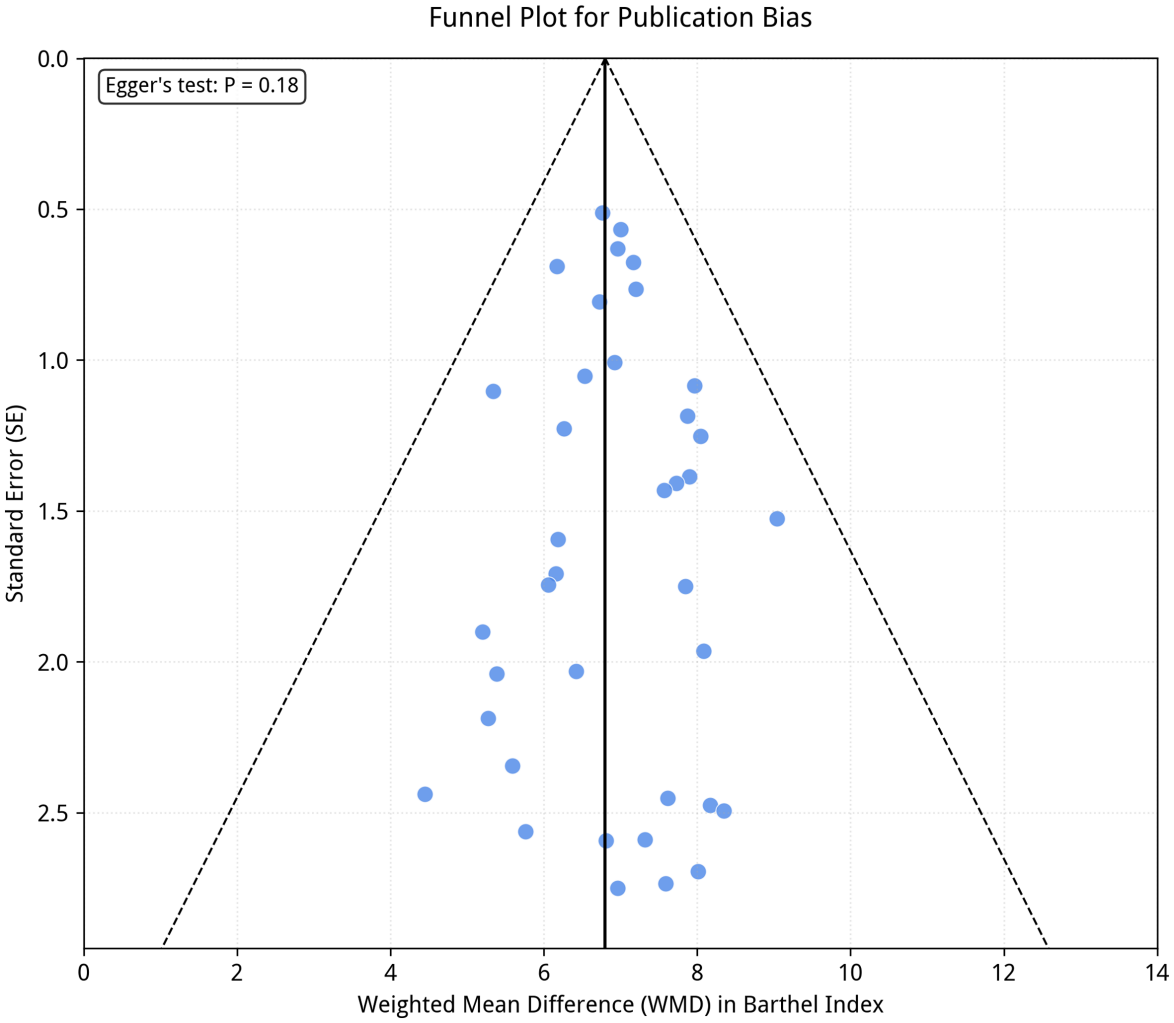
Funnel plot assessing publication bias for the primary outcome of Barthel Index improvement. The x-axis represents the weighted mean difference (WMD) of Barthel Index improvement, and the y-axis represents the standard error (SE) of the effect estimate. The solid vertical line indicates the pooled WMD. The dashed diagonal lines represent the 95% confidence interval limits for the effect size. No significant asymmetry was observed, consistent with the result of Egger’s regression test (*p* = 0.18).

Publication bias was visually evaluated using a funnel plot with the weighted mean difference (WMD) of Barthel Index improvement as the abscissa and standard error (SE) as the ordinate. The funnel plot for the primary outcome appeared symmetric, with study points distributed evenly on both sides of the pooled effect line (Figure 4). Egger’s regression test further confirmed no significant publication bias among the included studies (*p* = 0.18). These findings indicated that the pooled results were stable and less likely to be affected by publication bias.

### 3.8 Quality of evidence

The quality of evidence for the primary outcome (BI improvement) was rated as moderate for SSRIs and rTMS, high for CBT, and low for combined interventions according to the GRADE approach. Specific downgrading reasons included: (1) moderate risk of bias in 3/14 SSRIs studies (unclear randomization concealment); (2) imprecision for combined interventions (small sample size, *n* = 5, narrow confidence intervals not crossing the minimal clinically important difference); and (3) no serious inconsistency or indirectness for any intervention type. The detailed GRADE evidence profile is provided in Appendix S3.

The following discussion interprets these findings in the context of existing literature, explores underlying mechanisms, and highlights clinical implications and future research needs.

## 4 Discussion

### 4.1 Core findings

Our meta-analysis demonstrates that early emotional interventions confer significant functional benefits for stroke patients. More importantly, subgroup analyses reveal that this benefit is not uniform but is critically dependent on the timing and mechanism of the intervention, providing actionable insights for personalized clinical decision-making. The overall weighted mean difference (WMD) of 6.8 in Barthel Index (BI) scores reflects a clinically relevant enhancement in functional independence, supporting the potential value of integrating emotional interventions into early post-stroke care.

Notably, subgroup analysis uncovered key nuances that advance clinical decision-making. Interventions initiated within 2 weeks of stroke onset yielded substantially greater functional benefits (WMD = 10.3) compared to those started later (WMD = 5.8, *p* = 0.008), supporting the existence of a “therapeutic window” for emotional interventions in the acute post-stroke period, as illustrated in Figure 3 (18, 30). Among intervention types, cognitive behavioral therapy (CBT) (WMD = 8.2) and combined pharmacological-psychological approaches (WMD = 9.1) demonstrated the strongest effects (Figure 2), suggesting that multimodal strategies addressing both biological and psychological dimensions of emotional disorders may optimize outcomes (14, 21, 24, 27). In contrast, selective serotonin reuptake inhibitors (SSRIs) showed moderate but significant benefits (WMD = 4.2), particularly in younger ischemic stroke patients, aligning with prior observations of population-specific responses to pharmacological interventions (13, 15, 16).

For stroke type, early emotional interventions were effective for both ischemic (WMD = 7.1) and hemorrhagic stroke (WMD = 6.2), with no significant difference between subgroups (*p* = 0.42), indicating broad applicability across stroke subtypes. The beneficial effects persisted over long-term follow-up (≥ 6 months, WMD = 6.9), confirming that emotional interventions contribute to sustained functional recovery rather than transient symptom relief (3, 7).

The heterogeneity in functional benefits across intervention types and patient subgroups, as highlighted in our core findings, prompts a closer examination of the underlying mechanisms—particularly how different interventions modulate overlapping yet distinct pathways to drive functional recovery.

### 4.2 Mechanistic insights

**Figure 5:**
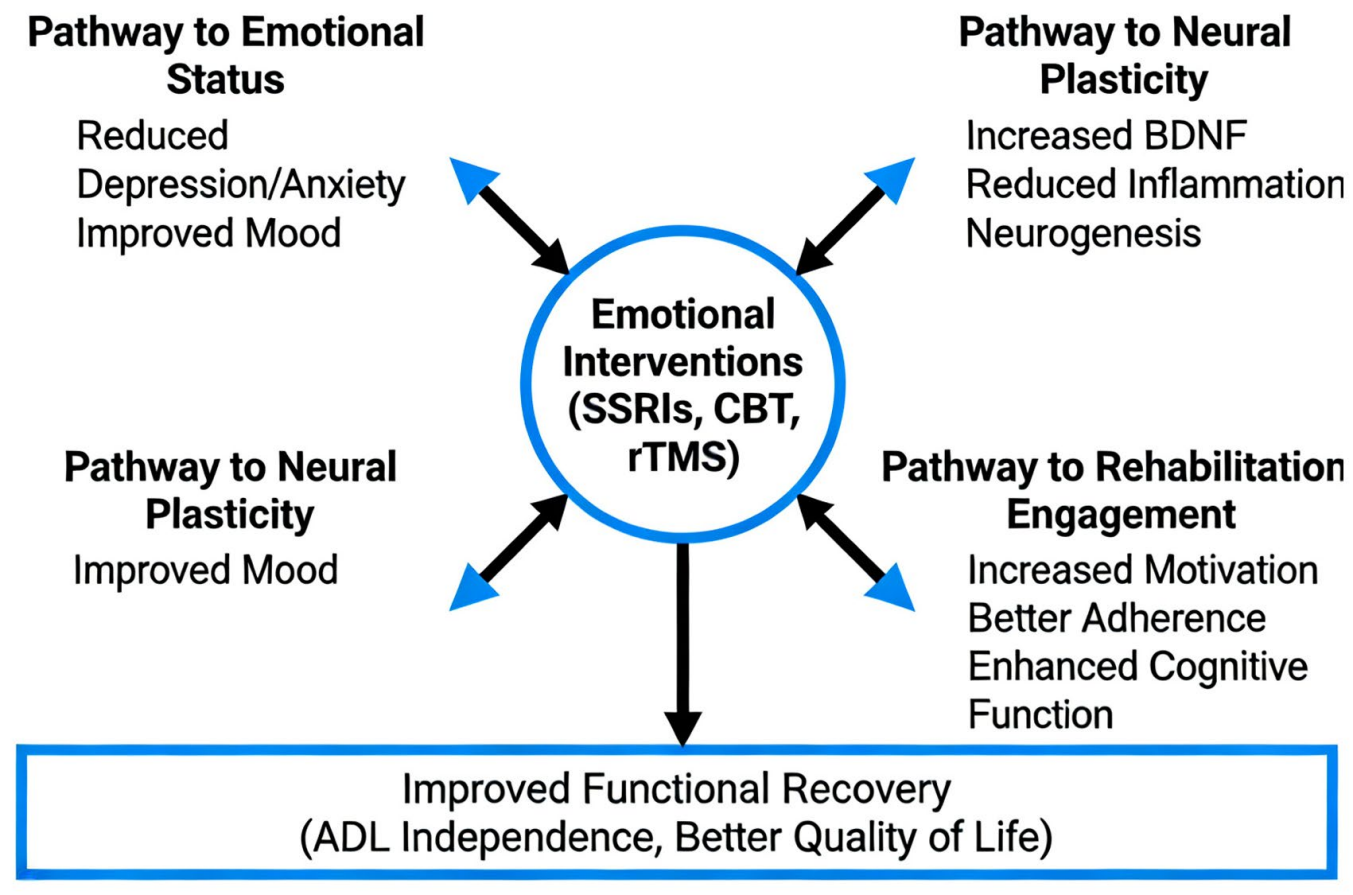
Mechanisms by which early emotional interventions improve functional recovery after stroke. Schematic illustration of proposed multifactorial mechanisms linking early emotional interventions to functional recovery post-stroke. Abbreviations: BDNF, brain-derived neurotrophic factor; CBT, cognitive behavioral therapy; rTMS, repetitive transcranial magnetic stimulation; SSRIs, selective serotonin reuptake inhibitors; ADL, activities of daily living.

The mechanisms underlying these benefits are multifactorial and interconnected, as illustrated in Figure 5. Emotional well-being enhances rehabilitation engagement by improving motivation and reducing fear-avoidance behaviors, which are critical for adherence to motor and cognitive training. This aligns with evidence that depression and anxiety impair executive function and attention—key cognitive domains for successful rehabilitation (1, 11).

Beyond behavioral pathways, interventions modulate biological processes critical for neurorecovery. Repetitive transcranial magnetic stimulation (rTMS) and CBT are thought to promote functional recovery by upregulating brain-derived neurotrophic factor (BDNF) and suppressing neuroinflammation. BDNF, a key mediator of neuroplasticity, is consistently reduced in post-stroke depression (PSD) patients, and interventions that restore BDNF levels correlate with improved functional outcomes (31–33). SSRIs, while primarily known for neurotransmitter regulation, also exert direct neuroprotective effects, including reduction of pro-inflammatory cytokines such as IL-6 and CRP, which contribute to secondary brain injury and functional decline (13, 31, 33).

These overlapping pathways—behavioral, neurochemical, and inflammatory—highlight why targeting emotional health translates to tangible functional gains beyond symptom relief. The superior efficacy of combined interventions (WMD = 9.1) may stem from their ability to concurrently modulate multiple pathways, addressing both the psychological and biological underpinnings of post-stroke emotional disorders (23, 27). This aligns with the multidisciplinary team (MDT) rehabilitation model, which integrates psychological support, pharmacological management, and neurorehabilitation—consistent with the core mission of rehabilitation medicine to deliver holistic, patient-centered care (18). The synergy of different intervention modalities in combined approaches mirrors the collaboration of neurologists, psychologists, therapists, and pharmacists in MDT teams, emphasizing that post-stroke recovery requires coordinated efforts across disciplines to address the complex interplay of emotional, physical, and cognitive impairments (11, 23).

### 4.3 Clinical implications

Our findings carry substantial implications for stroke neurorehabilitation practice. First, the high prevalence of post-stroke emotional disorders (20–50% for depression, 18–30% for anxiety) and their negative impact on rehabilitation engagement support the integration of routine emotional screening into acute and subacute neurorehabilitation protocols (1, 2, 18, 30). Implementing validated tools such as the Hospital Anxiety and Depression Scale (HADS) within 1 week of stroke onset could facilitate early identification and intervention, as delayed treatment is associated with diminished functional benefits (7, 30).

Second, subgroup analysis provides a framework for individualized intervention selection. CBT and combined interventions showed the largest effect sizes, suggesting they may be particularly useful when clinically feasible, while SSRIs demonstrated moderate but significant benefits (12, 16, 23, 27). For patients with contraindications to pharmacotherapy (e.g., bleeding risk), rTMS or acupuncture offers effective alternatives, expanding treatment options for complex cases (25, 26).

Third, from a health economic perspective, the durability of functional benefits observed in long-term follow-up suggests that early emotional interventions may yield substantial cost savings for healthcare systems (3, 7). By promoting sustained functional independence, these interventions can reduce the need for long-term care facilities, home care services, and readmissions related to functional decline—key considerations for policy-makers seeking cost-effective rehabilitation strategies (10, 18, 29). This aligns with the American Heart Association’s scientific statement emphasizing emotional health as a core component of value-based stroke care (18).

### 4.4 Bias control and methodological rigor

We addressed potential biases through rigorous methodological approaches to enhance the reliability of our findings. Heterogeneity across studies (I^2^ = 42% for the primary outcome) was explored through prespecified subgroup analyses, which identified intervention timing, type, and stroke subtype as key sources of variability (17). This targeted analysis reduced the impact of clinical and methodological heterogeneity, strengthening the interpretability of pooled results.

Risk of bias was systematically assessed using the Cochrane RoB 2 tool, with only studies of low or moderate bias included in the meta-analysis (17). We excluded studies with high risk of bias in randomization or missing outcome data, minimizing the influence of flawed study design. Sensitivity analyses excluding high-risk studies or small sample sizes (n < 50) confirmed the robustness of our results, with no significant changes in effect sizes.

Publication bias was minimized through comprehensive database searches (including both English and Chinese databases). Funnel plot analysis with weighted mean difference (WMD) as the abscissa and standard error (SE) as the ordinate demonstrated symmetric distribution, indicating no significant asymmetry (Figure 4; Egger’s test *p* = 0.18) (17). The quality of evidence was graded using the Grading of Recommendations Assessment, Development and Evaluation (GRADE) approach, providing transparency for clinical decision-makers regarding the strength of recommendations for each intervention (moderate for SSRIs and rTMS, high for CBT, low for combined interventions) (18).

### 4.5 Limitations

Several limitations of this review should be acknowledged to contextualize our findings. First, despite our comprehensive search strategy covering seven English and Chinese databases, some relevant studies may have been missed—particularly those published in non-English/Chinese languages (e.g., Spanish, German, Japanese) or gray literature (e.g., unpublished dissertations, conference proceedings), which could introduce selection bias. However, the inclusion of both English and Chinese databases enhances generalizability to diverse populations, mitigating this limitation to some extent.

Second, heterogeneity in intervention protocols was particularly prominent among combined interventions (*I*^2^ = 38%), which varied widely in component combinations (e.g., fluoxetine vs. sertraline paired with CBT vs. problem-solving therapy) and treatment durations (4–12 weeks), as detailed in Appendix S2 (23, 27). This internal heterogeneity may have influenced the pooled effect size for combined interventions, warranting caution in overinterpreting their relative superiority without standardized protocols. For other interventions (e.g., CBT duration, rTMS stimulation parameters), variability also exists, though subgroup analyses partially addressed this by stratifying by key variables (14, 25).

Third, the quality of evidence varied across intervention types, with combined interventions graded as low quality due to smaller sample sizes and methodological variability (23, 27). This reflects the relative novelty of combined approaches, highlighting the need for larger, well-designed trials to confirm their efficacy.

Fourth, we focused on functional outcomes and emotional symptoms, and did not explore secondary outcomes such as caregiver burden or healthcare utilization. Future reviews could expand the scope to include these outcomes, providing a more holistic assessment of intervention impact.

Fifth, we did not extract data on lesion location (e.g., lobar, subcortical, brainstem) because this information was inconsistently reported across the 38 included RCTs. Given that frontal and basal ganglia lesions are known to differentially affect the risk of post-stroke depression (8, 9), future individual-patient data meta-analyses should examine whether the efficacy of early emotional interventions varies by lesion location.

Sixth, the definition and intensity of “usual care” varied across the 38 included trials (e.g., differences in rehabilitation frequency, setting, and adjunctive therapies). This variability may have contributed to the observed heterogeneity (*I*^2^ = 42%) and could influence the generalizability of our pooled estimates.

### 4.6 Future research directions

Future research should address these limitations to strengthen the evidence base for early emotional interventions in stroke care. Large, well-designed head-to-head RCTs are needed to directly compare the efficacy of different intervention modalities (e.g., CBT vs. rTMS vs. combined approaches) and determine optimal timing, duration, and intensity (23, 24). These trials should standardize intervention protocols and outcome measures to reduce heterogeneity and improve comparability.

Studies investigating long-term outcomes (≥ 2 years) are essential to confirm the durability of functional benefits and assess cost-effectiveness (3, 7). Cost-effectiveness analyses would provide critical data for healthcare policy-makers, supporting the integration of emotional interventions into resource-limited settings (18, 30).

Mechanistic research should explore biomarkers such as BDNF, inflammatory cytokines, and gut microbiota composition to identify predictors of treatment response. This could enable precision medicine approaches, tailoring interventions to individual patients based on biological and clinical profiles.

Additionally, research focusing on underrepresented populations—including patients with severe stroke, comorbid cognitive impairment, or young stroke—would fill critical evidence gaps (7, 34, 35). Finally, implementation research is needed to evaluate strategies for integrating routine emotional screening and intervention into clinical practice, ensuring that evidence-based interventions reach patients in real-world settings (18, 30, 36).

## 5 Conclusion

Early emotional interventions significantly improve functional outcomes among stroke patients, with the greatest benefits observed when interventions are initiated within 2 weeks of stroke onset. Cognitive behavioral therapy and combined pharmacological-psychological interventions demonstrate the strongest effects on functional recovery, while SSRIs and rTMS offer valuable alternatives for specific patient subgroups. These findings support the integration of routine emotional screening and targeted interventions into standard stroke care protocols, with individualized selection of intervention modality based on stroke type, age, and patient preferences.

The multifactorial mechanisms underlying these benefits—including enhanced rehabilitation engagement, modulation of neural plasticity, and suppression of neuroinflammation—highlight the importance of addressing emotional health as an integral component of comprehensive stroke rehabilitation. Rigorous methodological approaches, including subgroup analyses and bias assessment, strengthen the reliability of our conclusions, though future research is needed to address remaining evidence gaps. By prioritizing early emotional intervention, clinicians can optimize functional recovery, improve quality of life, and reduce the long-term burden of stroke for patients and healthcare systems worldwide.

## Data Availability

The raw data supporting the conclusions of this article will be made available by the authors, without undue reservation.
