# Supplementary material for: Early emotional interventions for post-stroke functional prognosis: a systematic review and meta-analysis
Source: Front Neurol. 2026 Jul 2;17:1793682. doi: 10.3389/fneur.2026.1793682 (PMC13372643; doi:10.3389/fneur.2026.1793682)
Supplement: Supplementary file 1 [file Supplementary_File_1.docx]

**Appendix S1: PubMed Detailed Search Strategy**

**Final Search Strategy (November 30, 2025)**

((“Stroke”[Mesh] OR “Cerebrovascular Accident”[Mesh] OR “Brain Infarction”[Mesh] OR “Cerebral Hemorrhage”[Mesh] OR “Ischemic Stroke”[Title/Abstract] OR “Hemorrhagic Stroke”[Title/Abstract] OR “Stroke”[Title/Abstract] OR “CVA”[Title/Abstract] OR “Cerebrovascular Accident”[Title/Abstract] OR “脑梗死”[Title/Abstract] OR “中风”[Title/Abstract]) AND

(“Depression”[Mesh] OR “Anxiety”[Mesh] OR “Affective Symptoms”[Mesh] OR “Mood Disorders”[Mesh] OR “Post-Stroke Depression”[Title/Abstract] OR “PSD”[Title/Abstract] OR “Poststroke Depression”[Title/Abstract] OR “Post-Stroke Anxiety”[Title/Abstract] OR “Emotional Disorders”[Title/Abstract] OR “Psychological Distress”[Title/Abstract] OR “情绪障碍”[Title/Abstract] OR “抑郁”[Title/Abstract] OR “焦虑”[Title/Abstract]) AND

(“Early Intervention”[Mesh] OR “Psychological Intervention”[Mesh] OR “Drug Therapy”[Mesh] OR “Repetitive Transcranial Magnetic Stimulation”[Mesh] OR “Cognitive Behavioral Therapy”[Mesh] OR “SSRIs”[Title/Abstract] OR “Selective Serotonin Reuptake Inhibitors”[Title/Abstract] OR “CBT”[Title/Abstract] OR “rTMS”[Title/Abstract] OR “Early Treatment”[Title/Abstract] OR “Acute Phase”[Title/Abstract] OR “Subacute Phase”[Title/Abstract] OR “干预”[Title/Abstract] OR “心理治疗”[Title/Abstract] OR “药物治疗”[Title/Abstract]) AND

(“Functional Recovery”[Mesh] OR “Activities of Daily Living”[Mesh] OR “Barthel Index”[Title/Abstract] OR “Functional Independence Measure”[Title/Abstract] OR “FIM”[Title/Abstract] OR “Functional Outcome”[Title/Abstract] OR “Recovery of Function”[Title/Abstract] OR “日常生活活动能力”[Title/Abstract] OR “功能预后”[Title/Abstract])) AND

(“Randomized Controlled Trial”[pt] OR “Controlled Clinical Trial”[pt] OR “Randomized”[Title/Abstract] OR “Placebo”[Title/Abstract] OR “Clinical Trial”[pt] OR “RCT”[Title/Abstract])

**Limits Applied**:

- Publication date: All dates up to November 30, 2025
- Language: English and Chinese
- Study type: Clinical Trial, Randomized Controlled Trial
- Humans only
- Adults (19+ years)

**Additional Search Filters**:

- Excluded letters, editorials, and case reports
- Excluded studies with follow-up < 4 weeks

**Notes on Search Strategy**:

- **Language restriction**: The search was restricted to English and Chinese. This may introduce language bias, as relevant studies published in other languages (e.g., Spanish, German, Japanese) could have been missed. However, we included both major English databases and CNKI to cover a broad geographic and ethnic population. The potential impact is acknowledged in the main text Limitations (Section 4.5).
- **Database adaptation**: The above strategy was adapted for EMBASE (using EMTREE terms), Cochrane Library, Web of Science, CINAHL, PsycINFO, and CNKI. Full search strings for each database are available from the corresponding author upon request.
- **中文关键词**: Chinese terms (脑梗死, 中风, 情绪障碍, 抑郁, 焦虑, 干预, 心理治疗, 药物治疗, 日常生活活动能力, 功能预后) were included to capture publications in Chinese databases.
