# Supplementary material for: Early emotional interventions for post-stroke functional prognosis: a systematic review and meta-analysis
Source: Front Neurol. 2026 Jul 2;17:1793682. doi: 10.3389/fneur.2026.1793682 (PMC13372643; doi:10.3389/fneur.2026.1793682)
Supplement: Supplementary file 2 [file Supplementary_File_2.docx]

**Appendix S2: Data Extraction Form Template**

**Study Identification Information**

| **Field** | **Description** | **Example** |
| --- | --- | --- |
| Study ID | Unique identifier assigned by reviewers | STROKE-001 |
| First author | First author of the study | Smith J |
| Publication year | Year of publication | 2023 |
| Journal | Journal name | Stroke |
| Country | Country where the study was conducted | United States |
| Study design | Type of study design | Randomized controlled trial |
| Funding source | Source of funding for the study | National Institutes of Health |
| Conflict of interest | Any declared conflicts of interest | None declared |

**Patient Characteristics**

| **Field** | **Description** | **Example** |
| --- | --- | --- |
| Total sample size | Total number of participants | 200 |
| Intervention group size | Number of participants in intervention group | 100 |
| Control group size | Number of participants in control group | 100 |
| Mean age (years) | Mean age of participants | 65.2 |
| Age range (years) | Age range of participants | 45-85 |
| Gender distribution | Percentage of male/female participants | 55% male, 45% female |
| Stroke type | Type of stroke (ischemic/hemorrhagic/mixed) | 80% ischemic, 20% hemorrhagic |
| Stroke location (if reported) | Cerebral lobe or region affected | Frontal lobe (n=30), basal ganglia (n=45) |
| Time since stroke (days) | Mean time from stroke to intervention | 30 |
| Baseline BI score | Mean baseline Barthel Index score | 55 |
| Baseline HAMD score | Mean baseline Hamilton Depression Rating Scale score | 18 |
| Comorbidities | Presence of comorbid conditions | Hypertension (60%), Diabetes (30%) |

**Intervention Details**

| **Field** | **Description** | **Example** |
| --- | --- | --- |
| Intervention type | Type of emotional intervention | Cognitive behavioral therapy |
| Intervention name | Specific name of the intervention | CBT for post-stroke depression |
| Intervention provider | Professional providing the intervention | Psychologist |
| Intervention duration | Total duration of intervention | 12 weeks |
| Session frequency | Number of sessions per week | 1 session/week |
| Session duration (minutes) | Duration of each session | 60 |
| Total sessions | Total number of sessions | 12 |
| Intervention components | Key components of the intervention | Cognitive restructuring, behavioral activation |
| Adherence rate | Percentage of participants completing all sessions | 85% |
| Co-interventions in experimental group | Additional treatments received beyond the studied intervention | Both groups received identical usual care; emotional intervention was added |

**Control Group Details**

| **Field** | **Description** | **Example** |
| --- | --- | --- |
| Control type | Type of control condition | Usual care |
| Control description | Description of the control condition | Standard stroke rehabilitation |
| Usual care components | Specific elements of usual care (e.g., standard rehabilitation, medical management) | Standard stroke unit care including physiotherapy (30 min/day, 5 days/week), occupational therapy, and speech therapy as needed |
| Control duration | Duration of control condition | 12 weeks |
| Additional treatments | Any additional treatments provided | None |

**Outcome Measures**

| **Field** | **Description** | **Example** |
| --- | --- | --- |
| Primary outcome | Primary outcome measure | Barthel Index |
| Secondary outcomes | Secondary outcome measures | HAMD, SF-36, Adverse events |
| Follow-up time points | Time points for outcome assessment | 4 weeks, 12 weeks, 6 months |
| Outcome data - Intervention | Outcome data for intervention group | Mean BI = 75, SD = 10 |
| Outcome data - Control | Outcome data for control group | Mean BI = 65, SD = 12 |
| Statistical significance | P-value for between-group comparison | P = 0.02 |
| Effect size | Calculated effect size | Cohen's d = 0.8 |

**Quality Assessment**

| **Field** | **Description** | **Example** |
| --- | --- | --- |
| Randomization method | Method used for randomization | Computer-generated random sequence |
| Allocation concealment | Method of allocation concealment | Central randomization |
| Blinding | Blinding of participants, providers, outcome assessors | Single-blind (outcome assessors) |
| Attrition rate | Percentage of participants lost to follow-up | 10% |
| Intention-to-treat analysis | Whether ITT analysis was used | Yes |
| Co-intervention reporting | Whether co-interventions were described and balanced between groups | Yes, both groups received identical standard rehabilitation |
| Risk of bias | Overall risk of bias (low/some concerns/high) | Low risk |

**Additional Notes**

| **Field** | **Description** | **Example** |
| --- | --- | --- |
| Key findings | Key findings of the study | CBT significantly improved functional outcomes |
| Limitations | Study limitations | Small sample size, single center |
| Comments | Additional comments from reviewers | High adherence rate, well-designed trial |

**Footnotes**:

- Inter-rater reliability: Data extraction was performed independently by two reviewers. Agreement was excellent (Cohen‘s κ = 0.89). Discrepancies were resolved by discussion or consultation with a third reviewer.
