# Supplementary material for: Early emotional interventions for post-stroke functional prognosis: a systematic review and meta-analysis
Source: Front Neurol. 2026 Jul 2;17:1793682. doi: 10.3389/fneur.2026.1793682 (PMC13372643; doi:10.3389/fneur.2026.1793682)
Supplement: Supplementary file 3 [file Supplementary_File_3.docx]

**Appendix S3: GRADE Evidence Profile for Key Outcomes**

**Table S3.1: GRADE Evidence Profile for Barthel Index Improvement**

| **Domain** | **SSRIs (Ischemic Stroke)** | **CBT (All Stroke Types)** | **rTMS (10Hz Left DLPFC)** | **Combined Interventions** |
| --- | --- | --- | --- | --- |
| **Number of studies** | 7 RCTs | 10 RCTs | 7 RCTs | 8 RCTs |
| **Total participants** | 2,842 | 3,567 | 2,134 | 2,308 |
| **Effect size (95% CI)** | WMD = 4.2 (1.8~6.6) | WMD = 8.2 (5.7~10.7) | WMD = 6.5 (4.1~8.9) | WMD = 9.1 (6.5~11.7) |
| **I² statistic** | 45% | 46% | 43% | 38% |
| **Initial quality** | High | High | High | High |
| **Downgraded for risk of bias** | -1 (3 studies with some concerns) | 0 (all studies low risk or some concerns) | -1 (2 studies with some concerns) | -1 (3 studies with some concerns) |
| **Downgraded for inconsistency** | 0 (moderate heterogeneity) | 0 (moderate heterogeneity) | 0 (moderate heterogeneity) | 0 (low heterogeneity) |
| **Downgraded for imprecision** | 0 (CI does not include null) | 0 (CI does not include null) | 0 (CI does not include null) | -1 (smaller sample size) |
| **Downgraded for indirectness** | -1 (only ischemic stroke) | 0 (all stroke types) | 0 (all stroke types) | 0 (all stroke types) |
| **Downgraded for publication bias** | 0 (funnel plot symmetric) | 0 (funnel plot symmetric) | 0 (funnel plot symmetric) | 0 (funnel plot symmetric) |
| **Upgraded for large effect** | 0 (small effect size) | +1 (large effect size) | +1 (moderate effect size) | +1 (large effect size) |
| **Upgraded for dose-response** | 0 (not observed) | 0 (not formally tested) | 0 (not observed) | 0 (not formally tested) |
| **Upgraded for confounding** | 0 (no confounding) | 0 (no confounding) | 0 (no confounding) | 0 (no confounding) |
| **Final GRADE quality** | **Moderate** | **High** | **Moderate** | **Low** |

Footnotes for Table S3.1:

- *a* – Downgraded for risk of bias when ≥2 studies had some concerns (mostly due to lack of allocation concealment or blinding of outcome assessors).
- *b* – Heterogeneity I² < 50% was not considered serious inconsistency; no downgrade applied.
- *c* – Combined interventions downgraded to Low due to imprecision (small total sample size, n=5 studies in the primary analysis; note that 8 RCTs contributed to the combined group in the overall analysis, but for BI specifically only 5 had extractable data) and substantial variation in component combinations (e.g., SSRI + CBT vs. SSRI + problem‑solving therapy).
- *d* – Effect size threshold for upgrading: large effect defined as WMD ≥ 5.0 (moderate: 2.5–4.9).

**Table S3.2: GRADE Evidence Profile for Emotional Symptom Improvement**

| **Domain** | **SSRIs** | **CBT** | **rTMS** | **Combined Interventions** |
| --- | --- | --- | --- | --- |
| **Number of studies** | 8 RCTs | 10 RCTs | 7 RCTs | 8 RCTs |
| **Total participants** | 3,125 | 3,567 | 2,134 | 2,308 |
| **Effect size (95% CI)** | SMD = -0.61 (-0.83~-0.39) | SMD = -0.58 (-0.81~-0.35) | SMD = -0.52 (-0.76~-0.28) | SMD = -0.73 (-0.98~-0.48) |
| **I² statistic** | 42% | 38% | 45% | 32% |
| **Initial quality** | High | High | High | High |
| **Downgraded for risk of bias** | -1 (3 studies with some concerns) | 0 (all studies low risk or some concerns) | -1 (2 studies with some concerns) | -1 (3 studies with some concerns) |
| **Downgraded for inconsistency** | 0 (moderate heterogeneity) | 0 (low heterogeneity) | 0 (moderate heterogeneity) | 0 (low heterogeneity) |
| **Downgraded for imprecision** | 0 (CI does not include null) | 0 (CI does not include null) | 0 (CI does not include null) | 0 (CI does not include null) |
| **Downgraded for indirectness** | 0 (all stroke types) | 0 (all stroke types) | 0 (all stroke types) | 0 (all stroke types) |
| **Downgraded for publication bias** | 0 (funnel plot symmetric) | 0 (funnel plot symmetric) | 0 (funnel plot symmetric) | 0 (funnel plot symmetric) |
| **Upgraded for large effect** | 0 (moderate effect size)* | 0 (moderate effect size)* | 0 (small effect size) | 0 (moderate effect size) |
| **Upgraded for dose-response** | 0 (not observed) | 0 (not formally tested) | 0 (not observed) | 0 (not formally tested) |
| **Upgraded for confounding** | 0 (no confounding) | 0 (no confounding) | 0 (no confounding) | 0 (no confounding) |
| **Final GRADE quality** | **High** | **High** | **Moderate** | **High** |

Footnotes for Table S3.2:

- For SSRIs and CBT, SMD values of –0.61 and –0.58 are moderate effect sizes (≥0.5). According to GRADE, upgrading for large effect typically requires SMD > 0.8. Therefore, no upgrade was applied. The final High quality reflects the absence of serious limitations across other domains.

**Table S3.3: GRADE Evidence Profile for Safety Outcomes**

| **Domain** | **SSRIs** | **CBT** | **rTMS** | **Combined Interventions** |
| --- | --- | --- | --- | --- |
| **Number of studies** | 8 RCTs | 10 RCTs | 7 RCTs | 8 RCTs |
| **Total participants** | 3,125 | 3,567 | 2,134 | 2,308 |
| **Adverse events (RR, 95% CI)** | 1.15 (1.03~1.29) | 0.98 (0.85~1.13) | 1.02 (0.89~1.17) | 1.05 (0.92~1.20) |
| **Serious adverse events (RR, 95% CI)** | 1.22 (0.98~1.52) | 0.95 (0.78~1.16) | 0.98 (0.81~1.19) | 1.01 (0.84~1.21) |
| **Bleeding events (RR, 95% CI)** | 1.82 (1.13~2.93) | 0.97 (0.75~1.25) | 1.00 (0.78~1.28) | 1.03 (0.81~1.31) |
| **Initial quality** | High | High | High | High |
| **Downgraded for risk of bias** | -1 (3 studies with some concerns) | 0 (all studies low risk or some concerns) | -1 (2 studies with some concerns) | -1 (3 studies with some concerns) |
| **Downgraded for inconsistency** | 0 (low heterogeneity) | 0 (low heterogeneity) | 0 (low heterogeneity) | 0 (low heterogeneity) |
| **Downgraded for imprecision** | -1 (CI includes null for serious adverse events) | 0 (CI includes null but small effect) | 0 (CI includes null but small effect) | 0 (CI includes null but small effect) |
| **Downgraded for indirectness** | 0 (all stroke types) | 0 (all stroke types) | 0 (all stroke types) | 0 (all stroke types) |
| **Downgraded for publication bias** | 0 (funnel plot symmetric) | 0 (funnel plot symmetric) | 0 (funnel plot symmetric) | 0 (funnel plot symmetric) |
| **Upgraded for large effect** | None (harmful outcome) | 0 (no effect) | 0 (no effect) | 0 (no effect) |
| **Upgraded for dose-response** | None | None | None | None |
| **Upgraded for confounding** | 0 (no confounding) | 0 (no confounding) | 0 (no confounding) | 0 (no confounding) |
| **Final GRADE quality** | **Low** | **High** | **Moderate** | **Moderate** |

Footnotes for Table S3.3:

- For SSRIs, the increased risk of bleeding events (RR = 1.82) and the overall adverse events (RR = 1.15) indicate potential harm. In GRADE, harmful effects are not upgraded; they may be downgraded further. The final Low quality reflects imprecision and risk of bias concerns.
- For CBT, rTMS, and combined interventions, no significant increase in adverse events was observed.

Supplementary Table S3.4: Summary of GRADE Quality Ratings by Outcome and Intervention

| **Intervention** | **Functional Recovery (Barthel Index)** | **Emotional Symptoms** | **Safety** |
| --- | --- | --- | --- |
| **SSRIs** | Moderate | High | Low |
| **CBT** | High | High | High |
| **rTMS** | Moderate | Moderate | Moderate |
| **Combined Interventions** | Low | High | Moderate |

**GRADE Rating Definitions**:

- **High**: Further research is very unlikely to change our confidence in the estimate of effect.
- **Moderate**: Further research is likely to have an important impact on our confidence in the estimate of effect and may change the estimate.
- **Low**: Further research is very likely to have an important impact on our confidence in the estimate of effect and is likely to change the estimate.
- **Very low**: Any estimate of effect is very uncertain.

**Upgrading and Downgrading Rules Applied in This Review**:

- *Risk of bias*: Downgraded one level if ≥2 studies (or >25%) had “some concerns” in the RoB 2 tool.
- *Inconsistency*: Downgraded one level if I² ≥ 50% and the direction of effect varied.
- *Imprecision*: Downgraded one level if the 95% CI crossed the null for continuous outcomes or if the total sample size was <400.
- *Indirectness*: Downgraded one level if the population, intervention, or outcome differed substantially from the review question (e.g., only ischemic stroke).
- *Publication bias*: Downgraded one level if funnel plot asymmetry or Egger’s test P < 0.10.
- *Large effect*: Upgraded one level if WMD ≥ 5.0 (BI) or SMD ≥ 0.8 (emotional symptoms) and the effect was robust.
- *Dose‑response*: Upgraded one level if a clear gradient was reported in ≥2 studies (not applied due to insufficient evidence).
