# Supplementary material for: Early emotional interventions for post-stroke functional prognosis: a systematic review and meta-analysis
Source: Front Neurol. 2026 Jul 2;17:1793682. doi: 10.3389/fneur.2026.1793682 (PMC13372643; doi:10.3389/fneur.2026.1793682)
Supplement: Supplementary file 4 [file Supplementary_File_4.docx]

**Appendix S4. List of 38 included randomized controlled trials**

| **No.** | **First Author (Year)** | **Full Citation (abbreviated)** | **Intervention Type** | **Primary Outcome** |
| --- | --- | --- | --- | --- |
| 1 | AFFINITY Trial (2020) | Lancet Neurol. 2020;19(8):651-660. doi:10.1016/S1474-4422(20)30207-6 | Fluoxetine vs placebo | Functional recovery |
| 2 | Chollet F (2011) | Lancet Neurol. 2011;10(2):123-130. (FLAME) | Fluoxetine vs placebo | Motor recovery |
| 3 | EFFECTS Trial (2020) | Lancet Neurol. 2020;19(8):661-669. | Fluoxetine vs placebo | Functional independence |
| 4 | FOCUS Trial (2019) | Lancet. 2019;393(10168):265-274. | Fluoxetine vs placebo | Functional outcome |
| 5 | Kraglund KL (2018) | Stroke. 2018;49(11):2568-2576. (TALOS) | Citalopram vs placebo | Neurological function |
| 6 | Andersen G (1994) | Stroke. 1994;25(6):1099-1104. | Citalopram vs placebo | Depression + function |
| 7 | Almeida OP (2006) | J Clin Psychiatry. 2006;67(7):1104-1109. | Sertraline vs placebo | Depression prevention |
| 8 | Kim JS (2017) | Lancet Psychiatry. 2017;4(1):33-41. | Escitalopram vs placebo | Depression + neurological function |
| 9 | Jorge RE (2010) | Arch Gen Psychiatry. 2010;67(2):187-196. | Escitalopram vs placebo | Cognitive recovery |
| 10 | Murray V (2005) | J Clin Psychiatry. 2005;66(6):708-716. | Sertraline vs placebo | Depression + function |
| 11 | Rasmussen A (2003) | Psychosomatics. 2003;44(3):216-221. | Sertraline vs placebo | Depression prevention |
| 12 | Wiart L (2000) | Stroke. 2000;31(8):1829-1832. | Fluoxetine vs placebo | Depression + function |
| 13 | Fruehwald S (2003) | J Neurol. 2003;250(3):347-351. | Fluoxetine vs placebo | Depression |
| 14 | Yan N (2024) | BMC Psychiatry. 2024;24(1):365. | Escitalopram vs sertraline | Depression + function |
| 15 | Lincoln NB (2003) | Stroke. 2003;34(1):111-115. | CBT vs usual care | Depression + ADL |
| 16 | Mitchell PH (2009) | Stroke. 2009;40(9):3073-3078. | Psychosocial + antidepressant vs usual care | Depression |
| 17 | Robinson RG (2008) | JAMA. 2008;299(20):2391-2400. | Escitalopram / problem-solving therapy | Depression prevention + function |
| 18 | Hill K (2019) | BMC Neurol. 2019;19(1):128. | Problem-solving therapy vs volunteer support | Mood |
| 19 | Visser MM (2016) | Stroke. 2016;47(1):135-142. | Problem-solving therapy vs usual care | Coping + quality of life |
| 20 | Thomas SA (2019) | Health Technol Assess. 2019;23(47):1-176. (BEADS) | Behavioural activation vs usual care | Depression |
| 21 | Hoffmann T (2015) | Top Stroke Rehabil. 2015;22(2):116-126. | Brief psychological intervention vs usual care | Depression + anxiety |
| 22 | Niu Y (2022) | J Stroke Cerebrovasc Dis. 2022;31(2):106225. | Acceptance and Commitment Therapy (ACT) vs usual care | Depression prevention |
| 23 | Xiong J (2026) | Top Stroke Rehabil. 2026;33(2):221-233. | Solution-focused brief therapy + mindfulness vs usual care | Depression |
| 24 | Hordacre B (2021) | J Neurol. 2021;268(4):1474-1484. | rTMS (10 Hz) vs sham | Depression + function |
| 25 | Liu C (2024) | J Affect Disord. 2024;354:82-88. | Transcutaneous auricular vagus nerve stimulation vs sham | Depression |
| 26 | Gu SY (2017) | Brain Stimul. 2017;10(2):270-274. | rTMS (10 Hz) vs sham | Depression |
| 27 | Valiengo LC (2017) | J Neurol Neurosurg Psychiatry. 2017;88(2):170-175. | tDCS vs sham | Depression |
| 28 | Duan H (2023) | Int J Environ Res Public Health. 2023;20(2):930. | rTMS + mindfulness vs sham | Depression |
| 29 | Kazinczi C (2025) | BMC Neurol. 2025;25(1):38. | tDCS + inhibitory control training | Depression + anxiety |
| 30 | Bonin Pinto C (2019) | Neurorehabil Neural Repair. 2019;33(8):643-655. | Fluoxetine + rTMS vs placebo + sham | Motor recovery |
| 31 | Liu Y (2025) | Neuropsychiatr Dis Treat. 2025;21:917-925. | Acupuncture + escitalopram vs escitalopram alone | Depression + function |
| 32 | Sun YT (2015) | Zhongguo Zhen Jiu. 2015;35(2):119-122. | Acupuncture + fluoxetine vs fluoxetine | Depression |
| 33 | Yin ZL (2022) | Zhongguo Zhen Jiu. 2022;42(11):1216-1220. | Acupuncture + rTMS vs usual care | Depression |
| 34 | Acler M (2009) | J Neurol. 2009;256(7):1152-1158. | Sertraline vs placebo | Motor excitability |
| 35 | Robinson RG (2000) | Am J Psychiatry. 2000;157(3):351-359. | Nortriptyline / fluoxetine vs placebo | Depression + function |
| 36 | Karaiskos D (2012) | J Neuropsychiatry Clin Neurosci. 2012;24(3):349-353. | Duloxetine vs citalopram vs sertraline | Depression + anxiety + fatigue |
| 37 | Palumbo A (2022) | Am J Phys Med Rehabil. 2022;101(10):937-946. | Music therapy vs usual care | Depression |
| 38 | Reding MJ (1986) | Arch Neurol. 1986;43(8):763-765. | Trazodone vs placebo | Depression |
