# Supplementary material for: Early emotional interventions for post-stroke functional prognosis: a systematic review and meta-analysis
Source: Front Neurol. 2026 Jul 2;17:1793682. doi: 10.3389/fneur.2026.1793682 (PMC13372643; doi:10.3389/fneur.2026.1793682)
Supplement: Supplementary file 5 [file Supplementary_File_5.docx]

**Appendix S5. Detailed Characteristics of the 38 Included Randomized Controlled Trials**

| **Study (first author, year)** | **Sample size (I/C)** | **Mean age (years)** | **% male** | **Stroke type (% ischemic)** | **Intervention type** | **Timing (weeks post-stroke)** | **Follow-up (months)** | **Primary functional outcome** |
| --- | --- | --- | --- | --- | --- | --- | --- | --- |
| AFFINITY 2020 | 642/640 | 69.2 | 58% | 100% | SSRI (fluoxetine) | <2 | 6 | mRS |
| Chollet 2011 (FLAME) | 59/61 | 66.5 | 62% | 100% | SSRI (fluoxetine) | <1 | 3 | FIM |
| EFFECTS 2020 | 750/748 | 71.0 | 55% | 100% | SSRI (fluoxetine) | <2 | 6 | mRS |
| FOCUS 2019 | 1555/1555 | 70.2 | 54% | 100% | SSRI (fluoxetine) | <2 | 6 | mRS |
| Kraglund 2018 (TALOS) | 64/63 | 68.5 | 56% | 100% | SSRI (citalopram) | <1 | 3 | BI |
| Andersen 1994 | 33/33 | 71.0 | 52% | 100% | SSRI (citalopram) | <4 | 3 | BI |
| Almeida 2006 | 62/62 | 72.0 | 55% | 100% | SSRI (sertraline) | <4 | 12 | BI |
| Kim JS 2017 | 237/236 | 65.3 | 60% | 100% | SSRI (escitalopram) | <2 | 3 | mRS |
| Jorge 2010 | 60/60 | 66.8 | 57% | 100% | SSRI (escitalopram) | <4 | 3 | BI |
| Murray 2005 | 62/62 | 70.0 | 54% | 100% | SSRI (sertraline) | <8 | 12 | BI |
| Rasmussen 2003 | 70/70 | 69.0 | 53% | 100% | SSRI (sertraline) | <4 | 12 | BI |
| Wiart 2000 | 15/15 | 68.0 | 50% | 100% | SSRI (fluoxetine) | <4 | 3 | BI |
| Fruehwald 2003 | 26/26 | 68.0 | 55% | 100% | SSRI (fluoxetine) | <2 | 3 | BI |
| Yan 2024 | 45/45 | 66.0 | 55% | 100% | SSRI (escitalopram vs sertraline) | <4 | 3 | BI |
| Lincoln 2003 | 20/20 | 68.0 | 60% | 75% | CBT | <8 | 6 | BI |
| Mitchell 2009 | 50/50 | 67.5 | 55% | 80% | Combined (SSRI + psychosocial) | <2 | 12 | BI |
| Robinson 2008 | 62/61 | 65.0 | 58% | 70% | Combined (escitalopram + problem-solving) | <2 | 12 | BI |
| Hill 2019 | 60/60 | 69.0 | 56% | 100% | Psychological (problem-solving) | <8 | 6 | BI |
| Visser 2016 | 55/55 | 68.0 | 54% | 100% | Psychological (problem-solving) | <12 | 6 | BI |
| Thomas 2019 | 20/20 | 67.0 | 55% | 100% | Psychological (behavioural activation) | <8 | 6 | BI |
| Hoffmann 2015 | 30/30 | 66.0 | 60% | 100% | Brief psychological | <2 | 3 | BI |
| Niu 2022 | 35/35 | 68.0 | 52% | 80% | ACT | <4 | 6 | BI |
| Xiong 2026 | 40/40 | 67.0 | 55% | 75% | Solution-focused + mindfulness | <2 | 6 | BI |
| Hordacre 2021 | 15/15 | 65.0 | 53% | 100% | rTMS (10 Hz) | <12 | 1 | BI |
| Liu C 2024 | 30/30 | 66.5 | 55% | 100% | taVNS | <4 | 2 | HAMD |
| Gu 2017 | 10/10 | 64.0 | 60% | 100% | rTMS | >24 | 1 | BI |
| Valiengo 2017 | 20/20 | 63.0 | 55% | 100% | tDCS | <4 | 1 | BI |
| Duan 2023 | 38/38 | 66.0 | 50% | 100% | rTMS + mindfulness | <4 | 3 | BI |
| Kazinczi 2025 | 20/20 | 65.0 | 55% | 100% | tDCS + inhibitory training | <12 | 1 | BI |
| Bonin Pinto 2019 | 15/15 | 64.0 | 60% | 100% | Combined (fluoxetine + rTMS) | <4 | 1 | BI |
| Liu Y 2025 | 40/40 | 67.0 | 53% | 80% | Combined (acupuncture + escitalopram) | <4 | 3 | BI |
| Sun YT 2015 | 30/30 | 66.0 | 55% | 100% | Combined (acupuncture + fluoxetine) | <4 | 2 | BI |
| Yin ZL 2022 | 40/40 | 67.0 | 52% | 75% | Combined (acupuncture + rTMS) | <4 | 3 | BI |
| Acler 2009 | 16/16 | 67.0 | 56% | 100% | SSRI (sertraline) | <4 | 1 | Motor excitability |
| Robinson 2000 | 22/23 | 66.0 | 55% | 100% | Nortriptyline/fluoxetine | <4 | 3 | BI |
| Karaiskos 2012 | 30/30 | 65.0 | 54% | 100% | SSRI (duloxetine vs citalopram vs sertraline) | <4 | 3 | BI |
| Palumbo 2022 | 20/20 | 68.0 | 55% | 100% | Music therapy | <4 | 3 | BI |
| Reding 1986 | 17/17 | 66.0 | 50% | 100% | Trazodone | <4 | 1 | BI |

*Note: I/C = intervention/control; BI = Barthel Index; FIM = Functional Independence Measure; mRS = modified Rankin Scale; HAMD = Hamilton Depression Rating Scale; ACT = Acceptance and Commitment Therapy; taVNS = transcutaneous auricular vagus nerve stimulation; tDCS = transcranial direct current stimulation; rTMS = repetitive transcranial magnetic stimulation; CBT = cognitive behavioral therapy; SSRI = selective serotonin reuptake inhibitor.*
