# Supplementary material for: Early emotional interventions for post-stroke functional prognosis: a systematic review and meta-analysis
Source: Front Neurol. 2026 Jul 2;17:1793682. doi: 10.3389/fneur.2026.1793682 (PMC13372643; doi:10.3389/fneur.2026.1793682)
Supplement: Supplementary file 6 [file Supplementary_File_6.docx]

| **Section and Topic** | **Item #** | **Checklist item** | **Location where item is reported** |
| --- | --- | --- | --- |
| **TITLE** | | |  |
| Title | 1 | Identify the report as a systematic review. | Title page (Title explicitly includes "Systematic Review and Meta-Analysis") |
| **ABSTRACT** | | |  |
| Abstract | 2 | See the PRISMA 2020 for Abstracts checklist. | Abstract section (complete abstract covering background, methods, results, conclusions) |
| **INTRODUCTION** | | |  |
| Rationale | 3 | Describe the rationale for the review in the context of existing knowledge. | Introduction section (discussing PSEDs burden, evidence gaps, and clinical relevance) |
| Objectives | 4 | Provide an explicit statement of the objective(s) or question(s) the review addresses. | Introduction section (last paragraph, stating efficacy evaluation and subgroup exploration goals) |
| **METHODS** | | |  |
| Eligibility criteria | 5 | Specify the inclusion and exclusion criteria for the review and how studies were grouped for the syntheses. | Methods section (Section 2.1 "Study Design and Eligibility Criteria" with explicit inclusion/exclusion lists) |
| Information sources | 6 | Specify all databases, registers, websites, organisations, reference lists and other sources searched or consulted to identify studies. Specify the date when each source was last searched or consulted. | Methods section (Section 2.2 "Search Strategy" listing 7 databases; search up to November 30, 2025) |
| Search strategy | 7 | Present the full search strategies for all databases, registers and websites, including any filters and limits used. | Methods section (search terms described; full strategy in Appendix S1, referenced in Section 2.2) |
| Selection process | 8 | Specify the methods used to decide whether a study met the inclusion criteria of the review, including how many reviewers screened each record and each report retrieved, whether they worked independently, and if applicable, details of automation tools used in the process. | Methods section (Section 2.3 "Study Selection and Data Extraction" describing independent screening by two reviewers, third reviewer for disagreements) |
| Data collection process | 9 | Specify the methods used to collect data from reports, including how many reviewers collected data from each report, whether they worked independently, any processes for obtaining or confirming data from study investigators, and if applicable, details of automation tools used in the process. | Methods section (Section 2.3 "Data extraction was performed independently by two reviewers" using standardized form [Appendix S2]) |
| Data items | 10a | List and define all outcomes for which data were sought. Specify whether all results that were compatible with each outcome domain in each study were sought (e.g. for all measures, time points, analyses), and if not, the methods used to decide which results to collect. | Methods section (Section 2.3 listing outcomes: BI, FIM, mRS, HAMD, HAMA, SF-36/SS‑QOL, mortality) |
|  | 10b | List and define all other variables for which data were sought (e.g. participant and intervention characteristics, funding sources). Describe any assumptions made about any missing or unclear information. | Methods section (Section 2.3 extracting study characteristics, patient demographics, intervention details; no assumptions reported for missing data) |
| Study risk of bias assessment | 11 | Specify the methods used to assess risk of bias in the included studies, including details of the tool(s) used, how many reviewers assessed each study and whether they worked independently, and if applicable, details of automation tools used in the process. | Methods section (Section 2.4 "Quality Assessment" using Cochrane RoB 2 tool; independent assessment implied by consistent methodology) |
| Effect measures | 12 | Specify for each outcome the effect measure(s) (e.g. risk ratio, mean difference) used in the synthesis or presentation of results. | Methods section (Section 2.5 "Statistical Analysis" specifying WMD for continuous outcomes, RR for dichotomous outcomes, SMD for secondary emotional/quality of life outcomes) |
| Synthesis methods | 13a | Describe the processes used to decide which studies were eligible for each synthesis (e.g. tabulating the study intervention characteristics and comparing against the planned groups for each synthesis (item #5)). | Methods section (Section 2.5 linking subgroup analyses to eligibility criteria: intervention type, timing, stroke type, follow-up duration) |
|  | 13b | Describe any methods required to prepare the data for presentation or synthesis, such as handling of missing summary statistics, or data conversions. | Not explicitly reported; assumed integrated in statistical methods (Section 2.5) |
|  | 13c | Describe any methods used to tabulate or visually display results of individual studies and syntheses. | Methods section (mentioning forest plots [Figure 2] and subgroup analysis figures [Figures 1, 3, 4]) |
|  | 13d | Describe any methods used to synthesize results and provide a rationale for the choice(s). If meta-analysis was performed, describe the model(s), method(s) to identify the presence and extent of statistical heterogeneity, and software package(s) used. | Methods section (specifying random-effects model, I² statistic, Review Manager 5.4 and Stata 17.0) |
|  | 13e | Describe any methods used to explore possible causes of heterogeneity among study results (e.g. subgroup analysis, meta-regression). | Methods section (listing four prespecified subgroup analyses to explore heterogeneity) |
|  | 13f | Describe any sensitivity analyses conducted to assess robustness of the synthesized results. | Methods section (stating sensitivity analyses excluding high-risk bias or small sample size studies) |
| Reporting bias assessment | 14 | Describe any methods used to assess risk of bias due to missing results in a synthesis (arising from reporting biases). | Methods/Results section (Section 3.7 "Publication Bias" describing funnel plots and Egger's regression test) |
| Certainty assessment | 15 | Describe any methods used to assess certainty (or confidence) in the body of evidence for an outcome. | Results section (Section 3.8 "Quality of Evidence" using GRADE approach; detailed profile in Appendix S3) |
| **RESULTS** | | |  |
| Study selection | 16a | Describe the results of the search and selection process, from the number of records identified in the search to the number of studies included in the review, ideally using a flow diagram. | Results section (Section 3.1 "Study Selection" with numerical results; Figure 1 PRISMA flow diagram) |
|  | 16b | Cite studies that might appear to meet the inclusion criteria, but which were excluded, and explain why they were excluded. | Results section (exclusion counts and categories provided in Figure 1) |
| Study characteristics | 17 | Cite each included study and present its characteristics. | Results section (Sections 3.2 "Study Characteristics" and 3.3 "Types of Emotional Interventions"; individual study citations in References) |
| Risk of bias in studies | 18 | Present assessments of risk of bias for each included study. | Not reported for individual studies; general method described in Methods (Section 2.4); mentioned in sensitivity analysis (Section 3.6) |
| Results of individual studies | 19 | For all outcomes, present, for each study: (a) summary statistics for each group (where appropriate) and (b) an effect estimate and its precision (e.g. confidence/credible interval), ideally using structured tables or plots. | Results section (pooled results presented in meta‑analysis (Figure 2, Table 1)) |
| Results of syntheses | 20a | For each synthesis, briefly summarise the characteristics and risk of bias among contributing studies. | Results section (Sections 3.4 and 3.5 summarizing study subsets for each synthesis) |
|  | 20b | Present results of all statistical syntheses conducted. If meta-analysis was done, present for each the summary estimate and its precision (e.g. confidence/credible interval) and measures of statistical heterogeneity. If comparing groups, describe the direction of the effect. | Results section (primary outcome, secondary outcomes, subgroup analyses) with WMD/RR, 95% CI, I², and P‑values |
|  | 20c | Present results of all investigations of possible causes of heterogeneity among study results. | Results section (Section 3.5 "Subgroup Analyses" reporting results for all four heterogeneity sources) |
|  | 20d | Present results of all sensitivity analyses conducted to assess the robustness of the synthesized results. | Results section (Section 3.6 "Sensitivity Analyses" confirming result robustness) |
| Reporting biases | 21 | Present assessments of risk of bias due to missing results (arising from reporting biases) for each synthesis assessed. | Results section (Section 3.7 "Publication Bias" reporting symmetric funnel plots and Egger's test P=0.18) |
| Certainty of evidence | 22 | Present assessments of certainty (or confidence) in the body of evidence for each outcome assessed. | Results section (Section 3.8 "Quality of Evidence" reporting GRADE ratings for each intervention type) |
| **DISCUSSION** | | |  |
| Discussion | 23a | Provide a general interpretation of the results in the context of other evidence. | Discussion section (Section 4.1 "Core Findings" integrating results with prior evidence) |
|  | 23b | Discuss any limitations of the evidence included in the review. | Discussion section (Section 4.5 "Limitations" discussing evidence gaps and study variability) |
|  | 23c | Discuss any limitations of the review processes used. | Discussion section (Section 4.5 "Limitations" including search strategy and heterogeneity‑related limitations) |
|  | 23d | Discuss implications of the results for practice, policy, and future research. | Discussion section (Section 4.3 "Clinical Implications" and Section 4.6 "Future Research Directions") |
| **OTHER INFORMATION** | | |  |
| Registration and protocol | 24a | Provide registration information for the review, including register name and registration number, or state that the review was not registered. | Not registered (protocol registration unsuccessful due to network restrictions, reported in non‑anonymised Editor's copy) |
|  | 24b | Indicate where the review protocol can be accessed, or state that a protocol was not prepared. | No protocol prepared (reported in non‑anonymised Editor's copy) |
|  | 24c | Describe and explain any amendments to information provided at registration or in the protocol. | Not applicable (no registered protocol) |
| Support | 25 | Describe sources of financial or non-financial support for the review, and the role of the funders or sponsors in the review. | Reported in non‑anonymised Editor's copy; no specific grant from public/commercial/not‑for‑profit sectors |
| Competing interests | 26 | Declare any competing interests of review authors. | Reported in non‑anonymised Editor's copy; all authors declare no financial/non‑financial conflicts of interest |
| Availability of data, code and other materials | 27 | Report which of the following are publicly available and where they can be found: template data collection forms; data extracted from included studies; data used for all analyses; analytic code; any other materials used in the review. | Reported in non‑anonymised Editor's copy; datasets used/analyzed available from corresponding author on reasonable request |

*From:*  Page MJ, McKenzie JE, Bossuyt PM, Boutron I, Hoffmann TC, Mulrow CD, et al. The PRISMA 2020 statement: an updated guideline for reporting systematic reviews. BMJ 2021;372:n71. doi: 10.1136/bmj.n71. This work is licensed under CC BY 4.0. To view a copy of this license, visit <https://creativecommons.org/licenses/by/4.0/>
